# Prevalence and risk factors for murine typhus, scrub typhus and spotted fever group rickettsioses among adolescent and adult patients presenting to Yangon General Hospital, Yangon, Myanmar

**DOI:** 10.1111/tmi.70009

**Published:** 2025-07-17

**Authors:** Thomas R. Bowhay, Tin Ohn Myat, Win Thandar Oo, Hla Kye Mone, Katrina J. Sharples, Matthew T. Robinson, Mayfong Mayxay, Paul N. Newton, Stuart D. Blacksell, Ampai Tanganuchitcharnchai, James E. Ussher, David R. Murdoch, Wah Win Htike, John A. Crump

**Affiliations:** ^1^ Centre for International Health, University of Otago Dunedin New Zealand; ^2^ Department of Microbiology University of Medicine 1 Yangon Myanmar; ^3^ Department of Mathematics and Statistics Division of Sciences, University of Otago Dunedin New Zealand; ^4^ Department of Medicine Dunedin School of Medicine, Division of Health Sciences, University of Otago Dunedin New Zealand; ^5^ Lao‐Oxford‐Mahosot Hospital‐Wellcome Trust Research Unit (LOMWRU), Microbiology Laboratory, Mahosot Hospital Vientiane Lao PDR; ^6^ Centre for Tropical Medicine and Global Health, Nuffield Department of Medicine University of Oxford Oxford UK; ^7^ Institute of Research and Education Development (IRED), University of Health Sciences, Ministry of Health Vientiane Lao PDR; ^8^ Mahidol‐Oxford Tropical Research Medicine Unit, Faculty of Tropical Medicine, Mahidol University Bangkok Thailand; ^9^ Department of Microbiology and Immunology University of Otago Dunedin New Zealand; ^10^ Department of Pathology and Biomedical Science University of Otago Christchurch New Zealand

**Keywords:** endemic flea‐borne typhus, epidemiology, fever, Myanmar, *Orientia tsutsugamushi*, *Rickettsia typhi*, scrub typhus, spotted fever group rickettsiosis

## Abstract

**Objectives:**

To inform patient management and disease prevention, we sought to estimate the prevalence of, and identify risk factors for, scrub typhus, murine typhus, and spotted fever group rickettsioses (SFGR) among febrile patients presenting to hospital in Myanmar.

**Methods:**

We recruited patients ≥12 years old with fever ≥38°C among those seeking care at Yangon General Hospital from 5 October 2015 through 4 October 2016. Standardised clinical and risk factor assessments were conducted. Confirmed scrub typhus, murine typhus, and SFGR infections were defined as a positive polymerase chain reaction or ≥4‐fold rise in immunofluorescence assay antibody titre to *Orientia tsutsugamushi, Rickettsia typhi* or *Rickettsia honei* or *Rickettsia conorii*, respectively. Probable infection was defined as IgM titre ≥1:400 to *O. tsutsugamushi*, an IgM titre of ≥1:800 or IgG ≥1:1600 to *R. typhi* or an IgG titre of ≥1:200 to *R. honeii* or *R. conorii.* Univariate and multivariable logistic regression was used to identify associations.

**Results:**

Among 944 participants, the median (range) age was 37 (12–94) years, 444 (47.0%) were female, and 704 (74.6%) resided in rural areas. Among participants, 63 (6.7%) had confirmed or probable scrub typhus and 15 (1.6%) had confirmed or probable murine typhus. No SFGR infections were identified. The odds of confirmed or probable scrub typhus were lower among females than males (adjusted odds ratio [aOR] 0.5, *p* = 0.014), lower among those earning >300,000 Kyat per month compared with those earning less than 100,000 Kyat per month (aOR 0.28, *p* = 0.039), and higher among agricultural workers compared with others (aOR 2.9, *p* = 0.004).

**Conclusion:**

Scrub typhus was common among patients presenting with fever in Yangon, murine typhus was uncommon, and SFGR was not found. Empiric treatment of severe febrile illness should include an antimicrobial with activity against rickettsial diseases. Public health campaigns targeting agricultural workers are recommended.

## INTRODUCTION

Rickettsial diseases, or rickettsioses, are among the most common causes of non‐malarial fever in Southeast Asia, where scrub typhus and murine typhus predominate [1]. Spotted fever group rickettsioses (SFGR) have been reported in Southeast Asia and are likely under‐recognised in the region [2, 3].

During World War II, outbreaks of scrub typhus and murine typhus were identified among military personnel stationed in Myanmar [4, 5]. Since then, there have been few publications on non‐malaria febrile illness [1, 6]. A 2004–2006 study of pregnant women on the border of Thailand and Myanmar found that up to 10% of those presenting with fever had confirmed scrub typhus or murine typhus by positive polymerase chain reactions (PCR) or ≥4‐fold rise in serum antibody titres [7]. A seroprevalence study of rickettsial infection in Myanmar on samples collected in 2019 found evidence of recent or past scrub typhus, murine typhus, and SFGR infection in 19%, 5%, and 3% of patients, respectively [8]. Scrub typhus seropositivity was highest in northern Myanmar, and male gender and increasing age were identified as risk factors for scrub typhus seropositivity [8]. Elsewhere in Southeast Asia, living rurally and working in agriculture have been identified as risk factors for scrub typhus seropositivity [9]. Living in an urban environment was associated with murine typhus seropositivity [3, 9]. Livestock exposure has been independently associated with both murine typhus and SFGR seropositivity [3]. Little is known about the prevalence of rickettsial diseases or risk factors for infection among febrile patients presenting to hospitals in Myanmar.

We sought to estimate the prevalence of scrub typhus, murine typhus, and SFGR among febrile participants presenting to Yangon General Hospital (YGH) Yangon, Myanmar; to describe the clinical features of febrile participants with these infections; and to identify risk factors such as sociodemographic and environmental exposures for scrub typhus, murine typhus, and SFGR.

## METHODS

### Study design

We undertook a prospective observational hospital‐based surveillance study of causes of febrile illness at YGH in Myanmar that examined bloodstream infections, as well as other infectious diseases associated with fever. Detailed study methods have been published elsewhere [10, 11]. A related study estimates the incidence of rickettsial disease in the Yangon Region [11].

### Setting

Yangon is the former capital and largest city in Myanmar with a population of 5.16 million and is situated in the Yangon Region [12]. YGH is a 2000‐bedded tertiary referral hospital that provides care to inpatients and outpatients ≥12 years old and receives patients from the community as well as from hospitals throughout the country. Febrile patients are triaged in the Emergency Department for referral to the Medical Observation (MO) Unit for admission or outpatient management.

### Participants

Potential participants were identified prospectively among adolescent and adult patients at the MO Unit of YGH from 5 October 2015 through 4 October 2016. Adolescent and adult patients aged ≥12 years were eligible for enrolment if they had an oral temperature of ≥38°C.

### Clinical data

A standardised questionnaire was used by trained medical graduates to collect demographic, clinical history, physical examination, and risk factor data from consenting participants. This included data on personal information, residence, including whether they lived rurally, detailed medication history, and environmental, occupational, and animal exposures. Clinical notes were reviewed to collect information on the provisional diagnoses, treatment given in hospital, and discharge outcome including diagnosis and inpatient death. For each participant, the Glasgow Coma Scale (GCS) score [13] and quick Sequential Organ Failure Assessment (qSOFA) score were calculated [14].

### Sample collection

Each participant had 15 mL of venous blood collected aseptically. Eight to 10 mL of blood was inoculated into BacT/ALERT FA blood culture bottles (bioMérieux, Inc., Durham, NC, USA), 2 mL of haemoculture fluid (HCF) from negative blood cultures, and 1.5 mL of serum was stored at −70°C. Convalescent serum was collected 14–30 days after admission. Samples were shipped on dry ice to reference laboratories for further testing.

### Laboratory methods

#### Serologic testing for scrub typhus, murine typhus, and SFGR

Serology for the detection of scrub typhus, murine typhus, and SFGR was performed at Mahidol Oxford Tropical Medicine Research Unit (MORU), Bangkok, Thailand. Serum samples at a dilution of 1:100 were first screened by the MORU in‐house ELISA to detect IgM and IgG antibodies against scrub typhus using *Orientia tsutsugamushi* Gilliam, Karp, Kato, and TA716 antigens; murine typhus using *Rickettsia typhi* Wilmington strain antigens; and SFGR using *Rickettsia conorii* and *Rickettsia honei* antigens [8]. Whole‐cell antigens were used. A net optical density (OD) at 450 nm of ≥0.5 was used as a cut‐off for samples to proceed to indirect immunofluorescence assay (IFA) testing [8].

Samples positive by ELISA screening were serially 2‐fold titrated from 1:100 to 1:25,600 using IFA for *O. tsutsugamushi* using pooled Gilliam, Karp, Kato, and TA716 antigens; for *R. typhi* using Wilmington strain antigens; and for SFGR using *R. honei* and *R. conorii* antigens. The test was not performed on paired sera with an ELISA IgG static OD or single sera with ELISA IgG levels above the 90th centile of all tested samples in the cohort, as the static paired sera IgG results and high single sera IgG results were most likely due to the presence of antibodies from past infections [15, 16].

#### Molecular testing for scrub typhus, murine typhus, and SFGR

Nucleic acid amplification tests were performed for scrub typhus, murine typhus, and SFGR on the HCF of participants with negative blood cultures at the Lao‐Oxford‐Mahosot Hospital‐Wellcome Trust Research Unit Vientiane, Lao PDR [10, 17]. Deoxyribonucleic acid was extracted from the HCF sample using QIAGEN Mini kit (QIAGEN, Germany) and tested by real‐time PCR using 47 and 17 kDa gene targets, surface membrane proteins of *O. tsutsugamushi* and *Rickettsia*, respectively [18, 19]. Samples with cycle threshold (Ct) values <40 were recorded as positive. Samples positive for 17 kDa underwent PCR testing to the *R. typhi‐*specific *ompB* target [20]. The 17 kDa PCR products of 17‐kDa‐positive and *ompB*‐negative samples were sent for sequencing at Macrogen Korea, Seoul, Republic of Korea to identify *Rickettsia* spp. other than *R. typhi*.

### Case definitions

Confirmed scrub typhus was defined as a participant with: a ≥4‐fold rise in IFA IgM or IgG titre between acute and convalescent serum; or PCR positive for 47 kDa [21]. Probable scrub typhus was defined as a participant with an acute or convalescent serum IFA IgM titre of ≥1:400 [22]. Scrub typhus exposure, which included confirmed or probable cases, was defined as a participant with: a serum IFA IgG titre of ≥1:100; or PCR positive for 47 kDa [21, 23, 24].

Confirmed murine typhus was defined as a participant with: a ≥4‐fold rise in IFA IgM or IgG titre between acute and convalescent serum; or PCR positive for 17 kDa and *R. typhi*‐specific *ompB* gene [21]. Probable murine typhus was defined as a participant with an acute or convalescent serum IFA IgM titre of ≥1:800 or IgG ≥1:1600 [16]. Murine typhus exposure was defined as a participant with: a serum IFA IgG titre of ≥1:100; or PCR positive for 17 kDa and *R. typhi*‐specific o*mpB* gene [8].

Confirmed SFGR was defined as a participant with a: ≥4‐fold rise in IFA IgM or IgG titre between acute and convalescent sera; or PCR positive for 17‐kDa, negative for *R. typhi* specific *ompB*, and sequenced to identify the *Rickettsia* spp. that was in the SFGR group [25]. Probable SFGR was defined as a participant with an acute or convalescent serum IFA IgG titre of ≥1:200; or PCR positive for 17 kDa and negative for *R. typhi* specific *ompB* and not meeting the confirmed case definition [25]. SFGR exposure was defined as a participant with: a serum IFA IgG titre of ≥1:100; or PCR positive for 17 kDa, negative for *R. typhi* specific *ompB* and sequenced to identify the *Rickettsia* spp. that was in the SFGR group [8].

### Statistical methods

Demographic and clinical characteristics, clinical management and exposure histories of cases and non‐cases were initially compared using univariate analyses. For binary variables, logistic regression was used to estimate unadjusted odds ratios with 95% confidence intervals and *p*‐values. For continuous variables, cases and controls were compared using the Hodges–Lehmann median difference with a 95% confidence interval and a Wilcoxon rank sum test [26]. Seasonal variation was assessed by defining two climate periods: the wet period from May through October and the dry period from November through April [27]. The date of presentation was used for inclusion in each period. A high qSOFA score was defined as a value ≥2 [14]. Participants receiving azithromycin or a tetracycline during hospital admission were considered to have received an agent with activity against rickettsioses.

To inform variable selection and model building for scrub typhus, we constructed directed acyclic graphs (DAG) to represent visually causal assumptions using epidemiological, environmental, and demographic variables in our dataset in a browser plugin [28]. We used the DAG to formulate a multivariable risk‐factor model determining the final model through backwards selection and comparison of the Akaike information criterion [29].

### Research ethics

The study received ethics approval from Ethics Review Committees of University of Medicine 1, and the Department of Medical Research, Yangon, Myanmar, and the Human Ethics Committee of the University of Otago. We sought and obtained written informed consent from guardians or caregivers for patients aged 12–18 years, those who were illiterate, or were unconscious at presentation. For all others, written consent was sought and obtained from the patient.

## RESULTS

### Study population, testing, demographics, and clinical data

**FIGURE 1:**
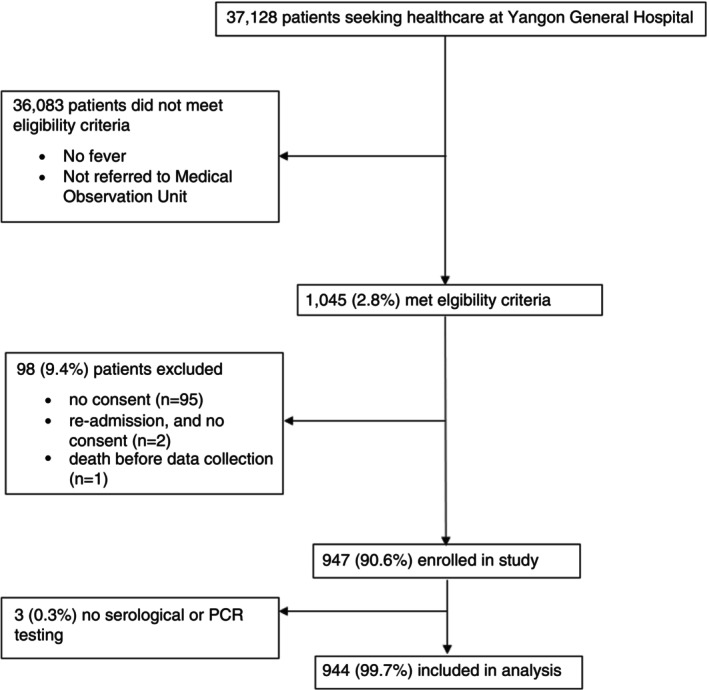
Study enrolment flow diagram, Yangon General Hospital, Myanmar, 2015–2016.

From 5 October 2015 through 4 October 2016, 37,128 patients were seen at the MO Unit of YGH. Of these patients, 1045 (2.8%) were eligible for inclusion in this study, and 947 (90.6%) consented and were enrolled (Figure 1). Three participants did not undergo any serological or PCR testing and were excluded from the analysis (Figure 1). Of 944 participants, 6 (0.6%) had no serological testing but had PCR testing and were included in the analysis. Of 938 participants who underwent serological testing, 367 (39.1%) had paired serum collected, and 571 (60.9%) had single serum alone collected. Of 944 participants, 690 (73.1%) culture‐negative HCF underwent PCR testing. Of 254 without PCR testing, 86 (33.9%) had paired serum collected. The demographic and clinical characteristics comparing participants with and without paired serum and with and without PCR testing are shown in Tables S1 and S2, respectively.

The median (range) age of the 944 participants was 37 (12–94) years, 444 participants (47.0%) were female and 704 (74.6%) lived in rural areas. The region of residence was Yangon Region for 671 (71.0%), Ayeyarwady Region for 107 (11.3%) and Bago Region for 102 (10.8%). Of the 944 participants, 928 (98.3%) were admitted to YGH, and 16 (1.7%) were treated as outpatients. No participants received an admission or discharge diagnosis of murine typhus, scrub typhus, or SFGR. Vital outcome data were missing for 38 (4.0%) of 944 participants.

### Scrub typhus

Of the 944 participants, 63 (6.7%) met the case definition for confirmed or probable scrub typhus; 37 of the 63 (58.7%) were confirmed cases, and 26 (41.2%) were probable cases. Of the 63 participants with confirmed or probable scrub typhus, 26 (41.3%) were diagnosed by PCR, 37 (58.7%) by serology, including 11 (17.5%) by both methods. Of the 944 participants, 85 (9.0%) met the case definition for scrub typhus exposure.

The findings of the univariate analyses are shown in Table 1. Of the 63 participants with confirmed or probable scrub typhus, 61 had mortality data available; two (3.3%) died compared to 62 (7.3%) of 881 participants without confirmed or probable scrub typhus and mortality data available (OR 0.43, *p* = 0.246). On multivariable analysis, being an agricultural worker (adjusted OR 3.9, *p* < 0.001) was associated with confirmed or probable scrub typhus. Being female (aOR 0.50, *p* = 0.014) and earning more than 300,000 Kyat per month (aOR 0.28, *p* = 0.039) was inversely associated with confirmed or probable scrub typhus (Table 2). The multivariable analysis for scrub typhus exposure is shown in Table 3.

**TABLE 1:** Features of febrile participants with and without confirmed or probable scrub typhus, and with and without scrub typhus exposure, Yangon General Hospital, Myanmar, 2015–2016.

	Confirmed or probable scrub typhus, *N* = 63		Not confirmed nor probable scrub typhus, *N* = 881					Scrub typhus exposure, *N* = 85		No scrub typhus exposure, *N* = 859				
Variable	*n*/*N*	(%)	*n*/*Nn*	(%)	OR	(95%CI)	*p*‐value^a^	*n*	(%)	*n*	(%)	OR	(95%CI)	*p*‐value^a^
Demographics														
Age, median (range) years	33	(13 to 75)	388	(12 to 94)	2.0	(−2 to 7)	0.344	33	(13 to 75)	38	(12 to 94)	3	(0 to 7)	0.112
Female sex	20	(31.8)	426	(48.1)	0.50	(0.29 to 0.87)	0.013	23	(37.7)	412	(48.0)	0.66	(0.41 to 1.0)	0.071
Presents during wet season^b^	34	(54.0)	489	(55.5)	0.94	(0.56 to 1.6)	0.813							
Rural	54	(85.7)	650	(73.8)	2.1	(1.04 to 4.4)	0.040	72	(84.7)	632	(73.6)	2.0	(1.1 to 3.7)	0.027
Agriculture worker	15	(23.8)	59	(6.7)	4.4	(2.3 to 8.2)	<0.001	20	(23.5)	54	(6.3)	4.6	(2.6 to 8.1)	<0.001
Income per month, Kyat^c^														
<100,000	30	(47.6)	332	(37.7)	Ref		Ref	43	(50.6)	319	(37.1)		Ref	Ref
100,000 to 300,000	30	(47.6)	417	(47.3)	0.80	(0.47 to 1.3)	0.396	36	(42.4)	411	(47.9)	0.65	(0.41 to 1.0)	0.070
>300,000	3	(4.8)	132	(15.0)	0.25	(0.08 to 0.83)	0.025	6	(7.1)	129	(15.0)	0.35	(0.14 to 0.83)	0.018
Presenting symptoms														
Days unwell prior to presentation, median (range)	10	(1 to 60)	77	(1 to 365)	−3	(−4 to −1)	0.004							
Days fever prior to presentation, median (range)	10	(1 to 25)	55	(1 to 30)	−3	(−4 to −1)	<0.001							
Chills and rigours	28	(44.4)	397	(45.1)	0.98	(0.58 to 1.6)	0.924							
Cough	21	(33.3)	259	(29.4)	1.2	(0.70 to 2.1)	0.509							
Headache	31	(49.2)	371	(42.1)	1.3	(0.80 to 2.2)	0.273							
Fever ≥1 month	5	(7.9)	186	(21.1)	0.32	(0.13 to 0.8)	0.017							
Presenting signs														
Conjunctival suffusion	6	(9.5)	16	(1.8)	5.7	(2.1 to 15.1)	<0.001							
Eschar	0		00											
Rash	3	(4.8)	42	(4.8)	1.0	(0.30 to 3.3)	0.998							
Lymphadenopathy	1	(1.6)	26	(3.0)	0.5	(0.07 to 4.0)	0.537							
Heart rate (median), BPM (range)	100	(28 to 120)	98	(50 to 170)	0	(−2 to 2)	0.857							
Lung crepitations	3	(4.8)	70	(8.0)	0.58	(0.18 to 1.9)	0.367							
Hepatomegaly	3	(4.8)	49	(5.6)	0.85	(0.26 to 2.8)	0.788							
Splenomegaly	1	(1.6)	23	(2.6)	0.60	(0.08 to 4.5)	0.622							
Meningism	1/63	(1.6)	23/879	(2.6)	0.60	(0.08 to 4.5)	0.620							
Environment														
Rodents around house	20	(31.8)	216	(24.4)	1.4	(0.83 to 2.5)	0.197	31	(36.5)	204	(23.8)	1.8	(1.2 to 3.0)	0.011
Rodent exposure at work	8	(12.7)	32	(3.6)	3.9	(1.70 to 8.8)	0.001	12	(14.1)	28	(3.3)	4.9	(2.4 to 10.0)	<0.001
Rodent exposure last month	5	(7.9)	40	(4.5)	1.8	(0.69 to 4.8)	0.228	9	(10.6)	36	(4.2)	2.7	(1.3 to 5.8)	0.011
Livestock contact last month	11	(17.5)	65	(7.4)	2.7	(1.3 to 5.3)	0.006	18	(21.2)	58	(6.8)	3.7	(2.1 to 6.7)	<0.001
Walking barefoot last month	10	(15.9)	52	(5.9)	3.0	(1.5 to 6.3)	0.003	13	(15.3)	49	(5.7)	3.0	(1.6 to 5.8)	0.001
Insect bite last month	0	(0)	7	(0.8)				0	(0)	7	(0.8)			
Insect bite mark last month	1	(1.6)	5	(0.6)	2.8	(0.33 to 24.6)	0.346	2	(2.4)	4	(0.5)	5.2	(0.93 to 28.5)	0.061
Clinical management														
Received doxycycline	0	(0)	2	(0.2)										
Received azithromycin	11	(17.5)	117	(13.3)	1.4	(0.70 to 2.7)	0.351							
Mortality	2/61	(3.3)	62/845	(7.3)	0.43	(0.10 to 1.8)	0.246							

Abbreviations: BPM, beats per minute; CI, confidence interval; OR, odds ratio.

^a^ For continuous variables, the difference of the median is shown, the test of significance is Wilcoxon rank sum, otherwise, the odds ratio and *p*‐value are by univariate logistic regression.

^b^ Wet season defined as November to April.

^c^ Equivalent in USD to <76.63, 73.63–220.89 and >220.89 in 2015 [30].

**TABLE 2:** Final multivariable logistic regression model of risk factors for confirmed or probable scrub typhus among febrile participants (*n* = 944), Yangon General Hospital, Myanmar 2015–2016.

Variables	Multivariable OR	(95% CI)	*p*‐value
Epidemiological/environmental			
Female sex	0.5	(0.29–0.88)	0.014
Agricultural worker	3.9	(2.0–7.3)	<0.001
Income per month, Kyat^a^			
<100,000	Ref	Ref	Ref
100,000–300,000	0.78	(0.46–1.3)	0.376
>300,000	0.28	(0.08–0.93)	0.039

Abbreviations: CI, confidence interval; OR, odds ratio.

^a^ Equivalent in USD to <76.63, 73.63–220.89 and >220.89 in 2015 [30].

**TABLE 3:** Final multivariable logistic regression model of risk factors for scrub typhus exposure among febrile participants (*n* = 944), Yangon General Hospital, Myanmar 2015–2016.

Variables	Multivariable OR	(95% CI)	*p*‐value
Epidemiological/environmental			
Age	0.99	(0.98–1.0)	0.084
Female sex	0.68	(0.42–1.0)	0.104
Agricultural worker	3.3	(1.7–6.2)	<0.001
Rodent exposure at work	2.4	(1.1–5.5)	0.035
Income per month, Kyat^a^			
<100,000	Ref	Ref	Ref
100,000–300,000	0.64	(0.39–1.0)	0.066
>300,000	0.39	(0.16–0.97)	0.042

Abbreviations: CI, confidence interval; OR, odds ratio.

^a^ Equivalent in USD to <76.63, 73.63–220.89 and >220.89 in 2015 [30].

### Murine typhus

Of 944 participants, 15 (1.6%) met the case definition for confirmed or probable murine typhus, 8 of the 15 (53.3%) were confirmed cases and 7 (46.7%) probable cases. Five of the 15 (33.3%) were diagnosed by PCR, 10 (66.7%) by serology, and none by both methods. Of the 944 participants, 35 (3.7%) met the case definition for murine typhus exposure. Of 15 participants with confirmed or probable murine typhus, 14 had mortality data; three (21.4%) died compared to 62 (6.8%) of 892 of those without confirmed or probable murine typhus (OR 3.7, *p* = 0.048). The reported causes of death for the three decedents were: systemic lupus erythematosus with nephropathy; acute viral illness; and severe aplastic anaemia.

The findings of the univariate analysis for murine typhus are shown in Table 4. No factors were found to be associated with murine typhus, but confidence intervals were wide due to the small numbers of cases. The odds of murine typhus exposure were lower among participants who lived rurally than those who did not (OR 0.50, *p* = 0.048).

**TABLE 4:** Features of febrile participants with and without confirmed or probable murine typhus, and with and without murine typhus exposure, Yangon General Hospital, Myanmar, 2015–2016.

Variable	Confirmed or probable murine typhus		Not confirmed nor probable murine typhus					Murine typhus exposure		No murine typhus exposure				
	*N* = 15		*N* = 929					*N* = 35		*N* = 909				
	*n*/*N*	(%)	*n*/*N*	(%)	OR	(95% CI)	*p*‐value^a^	*n*/*N*	(%)	*n*/*N*	(%)	OR	(95% CI)	*p*‐value^a^
Demographics														
Age, median (range) years	45	(14 to 72)	37	(12 to 94)	−1	(−9 to 8)	0.876	42	(14 to 83)	37	(12 to 94)			
Female sex	7	(46.7)	437	(47.0)	0.99	(0.35 to 2.7)	0.973	16/35	(35.7)	428	(47.1)	0.95	(0.48 to 1.9)	0.873
Presents during wet season^b^	6	(40.0)	517	(55.7)	0.53	(0.19 to 1.5)	0.233							
Rural	9	(60.0)	697	(74.8)	0.51	(0.18 to 1.4)	0.200	21/35	(60.0)	683	(75.1)	0.5	(0.25 to 0.99)	0.048
Agriculture worker	1	(6.7)	73	(7.9)	0.84	(0.11 to 6.5)	0.865	1	(2.9)	73	(8.0)	0.34	(0.05 to 2.5)	0.287
Income per month, Kyat^c^														
<100,000	4	(26.7)	358	(38.5)	Ref		Ref	13	(37.1)	349	(38.4)	Ref		Ref
100,000 to 300,000	9	(60)	438	(47.2)	1.8	(0.56 to 6.0)	0.314	14	(40.0)	433	(47.6)	0.87	(0.40 to 1.9)	0.718
>300,000	2	(13.3)	133	(14.3)	1.3	(0.24 to 7.4)	0.733	8	(22.9)	127	(14.0)	1.7	(0.68 to 4.2)	0.255
Presenting symptoms														
Days unwell prior to presentation, median (range)	8	(1 to 30)	7	(1 to 365)	−1	(−4 to 2)	0.467							
Days fever prior to presentation, median (range)	7	(1 to 25)	5	(1 to 30)	−2	(−4 to 0)	0.074							
Chills and rigours	10	(66.7)	415	(44.7)	2.5	(0.84 to 7.3)	0.103							
Cough	5	(33.3)	275	(29.6)	1.2	(0.40 to 3.5)	0.748							
Headache	7	(46.7)	395	(42.5)	1.2	(0.43 to 3.3)	0.752							
Fever ≥1 month	0	(0)	191	(20.6)										
Presenting signs														
Conjunctival suffusion	1	(6.7)	21	(2.3)	3.1	(0.39 to 24.6)	0.285							
Eschar	0		0											
Rash	1	(6.7)	44	(4.7)	1.4	(0.18 to 11.2)	0.727							
Lymphadenopathy	0	(0)	27	(2.9)										
Heart rate (median), BPM (range)	100	(82 to 130)	98	(28 to 170)	0	(−9 to 7)	0.715							
Lung crepitations	0	(0)	73	(7.9)										
Hepatomegaly	0	(0)	52	(5.6)										
Splenomegaly	0	(0)	24	(2.6)										
Meningism	0/15	(0)	24/927	(2.6)										
Environment														
Rodents around house	5	(33.3)	230	(24.8)	1.5	(0.51 to 4.5)	0.449	8	(22.9)	227	(25.0)	0.89	(0.40 to 2.0)	0.777
Rodent exposure at work	2	(13.3)	38	(4.1)	3.6	(0.79 to 16.6)	0.099	2	(5.7)	38	(4.2)	1.4	(0.32 to 6.0)	0.66
Rodent exposure last month	2	(13.3)	43	(4.6)	3.2	(0.69 to 14.5)	0.137	3	(8.6)	42	(4.6)	1.9	(0.57 to 6.6)	0.29
Livestock contact last month	1	(6.7)	75	(8.1)	0.81	(0.11 to 6.3)	0.843	1	(2.9)	75	(8.3)	0.33	(0.04 to 2.4)	0.274
Walking barefoot last month	0	(0)	62	(6.7)				0	(0)	62	(6.8)			
Insect bite last month	0	(0)	7	(0.8)				0	(0)	7	(0.8)			
Insect bite mark last month	0	(0)	6	(0.7)				0	(0)	6	(0.7)			
Clinical management														
Received doxycycline	0	(0)	2	(0.2)										
Received azithromycin	0	(0)	128	(13.8)										
Mortality	3/14	(21.4)	62/892	(6.8)	3.7	(1.0 to 13.7)	0.048							

Abbreviations: BPM, beats per minute; CI, confidence interval; OR, odds ratio.

^a^ For continuous variables, the difference of median is shown, the test of significance is Wilcoxon rank sum; otherwise, the odds ratio and *p*‐value are provided by univariate logistic regression.

^b^ Wet season defined as May to October.

^c^ Equivalent in USD to <76.63, 73.63–220.89 and >220.89 in 2015 [30].

### Scrub and murine typhus coinfection

Of 944 participants, four (0.4%) met the case definition for both confirmed scrub typhus and confirmed murine typhus. Scrub typhus was diagnosed by PCR in three and by serology in one. Murine typhus was diagnosed in all four by PCR. Of the three coinfections with mortality data available, one (33.3%) died.

### Spotted fever group rickettsioses

No participants met the case definition for confirmed SFGR. No 17 kDa‐positive and *ompB*‐negative samples had sufficient target concentration for successful sequencing. Five participants met the case definition for probable SFGR, all of which were diagnosed by PCR.

## DISCUSSION

We found that scrub typhus was a common cause of severe febrile illness, murine typhus was an uncommon cause, and no SFGR was identified among patients presenting with fever to YGH, Myanmar. Our study and a recent estimate of the incidence of scrub typhus and murine typhus in the Yangon region will contribute to understanding the burden and risk of these infections in Myanmar [11]. For scrub typhus, being female and earning more than 300,000 Kyat per month were associated with lower odds of disease, while being an agricultural worker was associated with higher odds of disease. For murine typhus exposure, living rurally had lower odds of infection. Notably, no participant was provisionally diagnosed or specifically treated for scrub typhus or murine typhus infection.

Our findings are consistent with those from countries neighbouring Myanmar. In a 2008–13 study of patients presenting to provincial hospitals with fever in Laos, using PCR and culture, 6.5% of patients were diagnosed with scrub typhus, and 0.5% with murine typhus [19]. Murine typhus, representing 6% of diagnoses in febrile participants in a study on the Thailand–Myanmar border, perhaps due to the outpatient setting of the study site where murine typhus, a milder illness than scrub typhus, may be more common [7]. Notably, the same study showed a similar prevalence of confirmed scrub typhus and murine typhus coinfection to that found at YGH. A 2013 review [31] identified four other instances of scrub typhus and murine typhus coinfection diagnosed using rigorous criteria of PCR or ≥4‐fold rise in IFA titre in China [32] and Laos [31]. Given the rigour of diagnostic methods, these likely represent true coinfections.

We identified just five probable SFGR infections in our study, all diagnosed by PCR. While SFGR is present [2, 8], our ability to detect SFGR may have been influenced by an unvalidated screening ELISA prior to IFA [33, 34]. Furthermore, low target concentration precluded sequencing of 17 kDa positive and *R. typhi* specific *ompB* negative samples, affecting our ability to speciate to the SFGR group.

For confirmed or probable scrub typhus, conjunctival suffusion was the only clinical sign associated with the disease [35]. Since conjunctival suffusion is also associated with a range of other febrile illnesses common in the study area, including leptospirosis, its diagnostic value is limited. No participants in our study had an eschar identified by the study team. This may be because the prevalence of eschar in scrub typhus is highly variable, including as low as 1% [36], or because they were overlooked despite the training of the clinical research team [37]. No presenting symptoms or signs were associated with confirmed or probable murine typhus, possibly related to the limited power due to the small number of cases in our study.

None of the participants in our study with scrub typhus and murine typhus were treated with doxycycline. The US Centers for Disease Control and Prevention recommends doxycycline as the first‐line treatment for both diseases [21]. Only 21.1% of confirmed cases of scrub typhus and 17.5% of confirmed or probable cases of scrub typhus received azithromycin, an effective alternative to doxycycline [38]. Azithromycin was almost always given in conjunction with other antimicrobials. The inclusion of doxycycline or azithromycin in combination with other empiric antimicrobials for severe febrile illness would ensure that there is coverage for scrub typhus in locations where scrub typhus is common. Of participants with confirmed or probable scrub typhus, 3.3% died, compared to the untreated mortality of 6.0% described in a systematic review [39]. No participants with murine typhus received azithromycin, a less effective agent for murine typhus than doxycycline [40]. Mortality associated with murine typhus was notably higher in our study than the 0.4% reported in the literature [41], perhaps due to co‐morbidities.

Despite our study being conducted in a hospital in the centre of Myanmar's largest city, most participants were from rural areas, and 29% were not from the Yangon Region. For scrub typhus, our univariate analysis findings are consistent with other research showing that living rurally is a risk factor for the disease [9]. Occupational exposure, in particular farming or agricultural work, has been linked to scrub typhus in many countries, including China, Laos, South Korea, and Thailand [9, 36]. Gender may be a surrogate for occupational exposures that vary from country to country [3, 37, 42]. In the multivariable analysis, high family income was protective for confirmed or probable scrub typhus. A 2010 community seroprevalence study in Vientiane City, Laos, found that low household income was an independent risk factor for scrub typhus seropositivity [9]. In the same study, poor sanitary conditions appeared to be an independent risk factor for scrub typhus [9]. Further research is needed to fully elucidate the connection between poverty and scrub typhus.

The findings from our univariate analysis were in keeping with previous research. Seeing rodents, the hosts of mites that are the main reservoir of scrub typhus, around the house, at work, or within the last month were all associated with scrub typhus exposure. Livestock exposure was also associated with scrub typhus exposure, which is consistent with findings from South Korea [43]. Livestock themselves are not thought to have a direct role in the lifecycle of *O. tsutsugamushi*, but animal fodder might attract rodents [44]. Walking barefoot may also increase the risk of mite attachment and feeding, leading to scrub typhus infection. In our univariate analysis, the odds of murine typhus exposure were significantly lower among those from rural areas than in non‐rural areas. This is consistent with research from both Laos and Thailand [3, 9].

Key limitations of our study include concerns for generalisability. We enrolled participants presenting to a tertiary hospital in a major city. As such, participants may not be representative of the general population in Myanmar. Only 38.8% of enrolled participants returned for convalescent serum collection, so comparison groups may have been contaminated by undiagnosed cases. While demographic characteristics were similar between those with and without paired serum, there were differences in some domains that may be a source of bias (Table S1). The testing strategy of excluding participants from IFA testing if a high or static IgG titre was detected by ELISA also limited our ability to diagnose cases of exposure. Mortality data were not available for all participants, and we relied on clinical records to establish causes of death. The diagnosis of rickettsial diseases is particularly difficult in participants who do not provide convalescent serum, as both PCR and acute serology have poor sensitivity [45]. Without an autopsy, mortality attributable to scrub typhus and murine typhus is likely underestimated.

We demonstrated that scrub typhus was a common cause of fever in patients presenting to hospital in Yangon and was associated with being an agriculture worker. Both being female and having a high income were protective for scrub typhus. We found scrub typhus difficult to predict based on clinical characteristics and environmental exposures. To avert fatal outcomes, we suggest the use of doxycycline or azithromycin in combination with other antimicrobials for empiric treatment of patients presenting with severe febrile illness or sepsis in Myanmar. Murine typhus appears to be uncommon among patients admitted to YGH, and no SFGR were identified. Scrub typhus preventive efforts could focus on agricultural workers increasing awareness of arthropod bites, avoiding vegetation favourable to mites, and using covered footwear, insect repellents, and permethrin‐treated clothing when possible [46]. The epidemiology of murine typhus remains poorly understood, and due to the milder nature of many infections, community‐based studies may be better suited to identify risk factors for infection and disease.

## CONFLICT OF INTEREST STATEMENT

The authors declare no conflicts of interest.

## Supporting information


**Table S1.** Comparison of clinical characteristics and environmental exposures between participants with and without paired serum for antibody testing for rickettsial diseases, Yangon General Hospital, Myanmar, 2015–2016.
**Table S2.** Comparison of clinical characteristics and environmental exposures between participants with and without PCR testing for rickettsial diseases, Yangon General Hospital, Myanmar, 2015–2016.

## References

[1] Acestor N , Cooksey R , Newton PN , Ménard D , Guerin PJ , Nakagawa J , et al. Mapping the aetiology of non‐malarial febrile illness in Southeast Asia through a systematic review‐Terra incognita impairing treatment policies. PLoS One. 2012;7:7. 10.1371/journal.pone.0044269 PMC3435412 22970193

[2] Parola P , Miller RS , McDaniel P , Telford SR , Rolain JM , Wongsrichanalai C , et al. Emerging rickettsioses of the Thai‐Myanmar border. Emerg Infect Dis. 2003;9:592–595. 10.3201/eid0905.020511 12737744 PMC2972759

[3] Chaisiri K , Tanganuchitcharnchai A , Kritiyakan A , Thinphovong C , Tanita M , Morand S , et al. Risk factors analysis for neglected human rickettsioses in rural communities in Nan province, Thailand: a community‐based observational study along a landscape gradient. PLoS Negl Trop Dis. 2022;16:1–16. 10.1371/journal.pntd.0010256 PMC8979453 35320277

[4] Mackie TT , Davis GE , Fuller HS , Knapp JA , Steinacker ML , Stager KE , et al. Observations on tsutsugamusiii disease (scrub typhus) in Assam and Burma: preliminary report. Am J Epidemiol. 1946;43:195–218. 10.1093/oxfordjournals.aje.a119063 20985364

[5] Kelly DJ , Richards AL , Temenak J , Strickman D , Dasch GA . The past and present threat of rickettsial diseases to military medicine and international public health. Clin Infect Dis. 2002;34:S145–S169. 10.1086/339908 12016590

[6] Shrestha P , Roberts T , Homsana A , Myat TO , Crump JA , Lubell Y , et al. Febrile illness in Asia: gaps in epidemiology, diagnosis and management for informing health policy. Clin Microbiol Infect. 2018;24:815–826. 10.1016/j.cmi.2018.03.028 29581051

[7] Mcgready R , Ashley EA , Wuthiekanun V , Tan SO , Pimanpanarak M , Viladpai‐Nguen SJ , et al. Arthropod borne disease: the leading cause of fever in pregnancy on the Thai‐Burmese border. PLoS Negl Trop Dis. 2010;4:4. 10.1371/journal.pntd.0000888 PMC2982829 21103369

[8] Elders PND , Swe MMM , Phyo AP , McLean ARD , Lin HN , Soe K , et al. Serological evidence indicates widespread distribution of rickettsioses in Myanmar. Int J Infect Dis. 2021;103:494–501. 10.1016/j.ijid.2020.12.013 33310022 PMC7862081

[9] Vallé J , Thaojaikong T , Moore CE , Phetsouvanh R , Richards AL , Souris M , et al. Contrasting spatial distribution and risk factors for past infection with scrub typhus and murine typhus in Vientiane city, Lao PDR. PLoS Negl Trop Dis. 2010;4:1–10. 10.1371/journal.pntd.0000909 PMC2998433 21151880

[10] Myat TO , Oo KM , Mone HK , Htike WW , Biswas A , Hannaway RF , et al. A prospective study of bloodstream infections among febrile adolescents and adults attending Yangon general hospital, Yangon, Myanmar. PLoS Negl Trop Dis. 2020;14:1–22. 10.1371/journal.pntd.0008268 PMC7217485 32352959

[11] Oo WT , Bowhay TR , Myat TO , Htike WW , Lwin KT , Blacksell SD , et al. Estimating scrub typhus and murine typhus incidence among adolescents and adults in Yangon, Myanmar. Trop Med Int Heal. 2025;30:978–986. 10.1111/tmi.70010 PMC12401641 40693720

[12] Department of Population Ministry of Immigration and Population . The 2014 Myanmar population and housing census. 2015. Available from: https://unstats.un.org/unsd/demographic-social/census/documents/Myanmar/MMR-2015-05.pdf

[13] Sternbach GL . The Glasgow coma scale. J Emerg Med. 2000;19:67–71. 10.1016/S0736-4679(00)00182-7 10863122

[14] Seymour CW , Liu VX , Iwashyna TJ , Brunkhorst FM , Rea TD , Scherag A , et al. Assessment of clinical criteria for sepsis for the third international consensus definitions for sepsis and septic shock (sepsis‐3). JAMA. 2016;315:762–774. 10.1001/jama.2016.0288 26903335 PMC5433435

[15] Varghese GM , Rajagopal VM , Trowbridge P , Purushothaman D , Martin SJ . Kinetics of IgM and IgG antibodies after scrub typhus infection and the clinical implications. Int J Infect Dis. 2018;71:53–55. 10.1016/j.ijid.2018.03.018 29653201 PMC5985369

[16] Phakhounthong K , Mukaka M , Dittrich S , Tanganuchitcharnchai A , Day NPJ , White LJ , et al. The temporal dynamics of humoral immunity to *Rickettsia typhi* infection in murine typhus patients. Clin Microbiol Infect. 2020;26:781.e9–781.e16. 10.1016/j.cmi.2019.10.022 PMC7284305 31678231

[17] Oo WT , Myat TO , Htike WW , Ussher JE , Murdoch DR , Lwin KT , et al. Incidence of typhoid and paratyphoid fevers among adolescents and adults in Yangon, Myanmar. Clin Infect Dis. 2019;68:S124–S129. 10.1093/cid/ciy1109 30845332 PMC6405279

[18] Jiang J , Chan T‐C , Temenak JJ , Dasch GA , Ching W‐M , Richards AL . Development of a quantitative real‐time polymerase chain reaction assay specific for *Orientia tsutsugamushi* . Am J Trop Med Hyg. 2004;70:351–356. 15100446

[19] Mayxay M , Castonguay‐Vanier J , Chansamouth V , Dubot‐Pérès A , Paris DH , Phetsouvanh R , et al. Causes of non‐malarial fever in Laos: a prospective study. Lancet Glob Health. 2013;1:e46. 10.1016/S2214-109X(13)70008-1 24748368 PMC3986032

[20] Henry KM , Jiang J , Rozmajzl PJ , Azad AF , Macaluso KR , Richards AL . Development of quantitative real‐time PCR assays to detect *Rickettsia typhi* and *Rickettsia felis*, the causative agents of murine typhus and flea‐borne spotted fever. Mol Cell Probes. 2007;21:17–23. 10.1016/j.mcp.2006.06.002 16893625

[21] Centers for Disease Control and Prevention . Typhus fevers. Clinical overview of murine typhus. Atlanta, GA: Centers for Disease Control and Prevention [Internet]; 2024. Available from: https://www.cdc.gov/typhus/hcp/clinical‐overview/clinical‐overview‐of‐murine‐typhus.html?CDC_AAref_Val=https://www.cdc.gov/typhus/healthcare‐providers/index.html

[22] Lim C , Blacksell SD , Laongnualpanich A , Kantipong P , Day NPJ , Paris DH , et al. Optimal cutoff titers for indirect immunofluorescence assay for diagnosis of scrub typhus. J Clin Microbiol. 2015;53:3663–3666. 10.1128/JCM.01680-15 26354819 PMC4609688

[23] Kim DM , Lee YM , Back JH , Yang TY , Lee JH , Song HJ , et al. A serosurvey of *Orientia tsutsugamushi* from patients with scrub typhus. Clin Microbiol Infect. 2010;16:447–451. 10.1111/j.1469-0691.2009.02865.x 19778303

[24] Elders PND , Dhawan S , Tanganuchitcharnchai A , Phommasone K , Chansamouth V , Day NPJ , et al. Diagnostic accuracy of an in‐house Scrub Typhus enzyme linked immunoassay for the detection of IgM and IgG antibodies. PLoS Negl Trop Dis. 2020;14:e0008858. 10.1371/journal.pntd.0008858 33284807 PMC7746293

[25] Centers for Disease Control and Prevention . Spotted fever rickettsiosis (including Rocky Mountain spotted fever) (SFR, including RMSF) 2020 case definition. Atlanta, GA: Centers for Disease Control and Prevention [Internet]; 2020 [cited 2022 Oct 19]. Available from: https://ndc.services.cdc.gov/case‐definitions/spotted‐fever‐rickettsiosis‐2020/

[26] Newson R . Parameters behind “nonparametric” statistics: Kendall's tau, Somers' D and median differences. Stata J. 2002;2:45–64. 10.1177/1536867x0200200103

[27] MoNREC . Myanmar Climate Change Strategy and Action Plan. Nay Pyi Taw, Myanmar: The Republic of the Union of Myanmar; 2017. Available from: https://policy.asiapacificenergy.org/sites/default/files/MCCSAP-Feb-Version.pdf

[28] Textor J , van der Zander B , Gilthorpe MS , Liśkiewicz M , Ellison GT . Robust causal inference using directed acyclic graphs: the R package “dagitty”. Int J Epidemiol. 2016;45:1887–1894. 10.1093/ije/dyw341 28089956

[29] Burnham KP , Anderson DR . Information and likelihood theory: a basis for model selection and inference. Model selection and multimodel inference. 2nd ed. New York, NY: Springer New York; 2002. p. 49–97. 10.1007/978-0−387-22456-5_2

[30] Central Bank of Myanmar . Reference exchange rate – history. 2022 [cited 2022 Sep 21]. Available from: https://forex.cbm.gov.mm/index.php/fxrate/history

[31] Phommasone K , Paris DH , Anantatat T , Castonguay‐Vanier J , Keomany S , Souvannasing P , et al. Concurrent infection with murine typhus and scrub typhus in southern Laos—the mixed and the unmixed. PLoS Negl Trop Dis. 2013;7:e2163. 10.1371/journal.pntd.0002163 24009783 PMC3757080

[32] Zhang L , Li X , Zhang D , Zhang J , Di Y , Luan M , et al. Molecular epidemic survey on co‐prevalence of scrub typhus and marine typhus in Yuxi city, Yunnan province of China. Chin Med J. 2007;120:1314–1318. Available from: http://www.ncbi.nlm.nih.gov/pubmed/17711736 17711736

[33] Robinson MT , Satjanadumrong J , Hughes T , Stenos J , Blacksell SD . Diagnosis of spotted fever group *Rickettsia* infections: the Asian perspective. Epidemiol Infect. 2019;147:e286. 10.1017/S0950268819001390 31587667 PMC6805790

[34] Paris DH , Dumler JS . State of the art of diagnosis of rickettsial diseases: the use of blood specimens for diagnosis of scrub typhus, spotted fever group rickettsiosis, and murine typhus. Curr Opin Infect Dis. 2016;29:433–439. 10.1097/QCO.0000000000000298 27429138 PMC5029442

[35] Palm TA . Some account of a disease called “Shima‐Mushi,” or “Island‐insect disease,” by the natives of Japan; peculiar, it is believed, to that country, and hitherto not described. Edinb Med J. 1878;24:128–132. PMC5317505 29640208

[36] Xu G , Walker DH , Jupiter D , Melby PC , Arcari CM . A review of the global epidemiology of scrub typhus. PLoS Negl Trop Dis. 2017;11:e0006062. 10.1371/journal.pntd.0006062 29099844 PMC5687757

[37] Kim DM , Kyung JW , Chi YP , Ki DY , Hyong SK , Tae YY , et al. Distribution of eschars on the body of scrub typhus patients: a prospective study. Am J Trop Med Hyg. 2007;76:806–809. 10.4269/ajtmh.2007.76.806 17488895

[38] Lee SC , Cheng YJ , Lin CH , Te Lei W , Chang HY , Lee MD , et al. Comparative effectiveness of azithromycin for treating scrub typhus. Medicine. 2017;96:e7992. 10.1097/MD.0000000000007992 28885357 PMC6392745

[39] Taylor AJ , Paris DH , Newton PN . A systematic review of mortality from untreated scrub typhus (*Orientia tsutsugamushi*). PLoS Negl Trop Dis. 2015;9:e0003971. 10.1371/journal.pntd.0003971 26274584 PMC4537241

[40] Newton PN , Keolouangkhot V , Lee SJ , Choumlivong K , Sisouphone S , Choumlivong K , et al. A prospective, open‐label, randomized trial of doxycycline versus azithromycin for the treatment of uncomplicated murine typhus. Clin Infect Dis. 2019;68:738–747. 10.1093/cid/ciy563 30020447 PMC6376095

[41] Doppler JF , Newton PN . A systematic review of the untreated mortality of murine typhus. PLoS Negl Trop Dis. 2020;14:1–13. 10.1371/journal.pntd.0008641 PMC7515178 32925913

[42] Kweon SS , Choi JS , Lim HS , Kim JR , Kim KY , Ryu SY , et al. Rapid increase of scrub typhus, South Korea, 2001–2006. Emerg Infect Dis. 2009;15:1127–1129. 10.3201/eid1507.080399 19624938 PMC2744253

[43] Kim DS , Acharya D , Lee K , Yoo SJ , Park JH , Lim HS . Awareness and work‐related factors associated with scrub typhus: a case‐control study from South Korea. Int J Environ Res Public Health. 2018;15:15. 10.3390/ijerph15061143 PMC6025028 29865144

[44] Traub R , Wisseman CL . Ecological considerations in scrub typhus. 1. Emerging concepts. Bull World Health Organ. 1968;39:209–218. 5303404 PMC2554547

[45] Paris DH , Blacksell SD , Nawtaisong P , Jenjaroen K , Teeraratkul A , Chierakul W , et al. Diagnostic accuracy of a loop‐mediated isothermal PCR assay for detection of *Orientia tsutsugamushi* during acute scrub typhus infection. PLoS Negl Trop Dis. 2011;5:e1307. 10.1371/journal.pntd.0001307 21931873 PMC3172190

[46] Centers for Disease Control and Prevention . Typhus fevers. Clinical overview of scrub typhus. Atlanta, GA: Centers for Disease Control and Prevention [Internet]; 2024. Available from: https://www.cdc.gov/typhus/hcp/clinical‐overview/clinical‐overview‐of‐scrub‐typhus.html?CDC_AAref_Val=https://www.cdc.gov/typhus/healthcare‐providers/index.html

