# Supplementary material for: Prevalence and risk factors for murine typhus, scrub typhus and spotted fever group rickettsioses among adolescent and adult patients presenting to Yangon General Hospital, Yangon, Myanmar
Source: Trop Med Int Health. 2025 Jul 17;30(9):966–77. doi: 10.1111/tmi.70009 (PMC12401629; doi:10.1111/tmi.70009)
Supplement: Supplementary file 1 — Table S1. Comparison of clinical characteristics and environmental exposures between participants with and without paired serum for antibody testing for rickettsial diseases, Yangon General Hospital, Myanmar, 2015–2016. Table S2. Comparison of clinical characteristics and environmental exposures between participants with and without PCR testing for rickettsial diseases, Yangon General Hospital, Myanmar, 2015–2016. [file TMI-30-966-s001.docx]

**Table S1. Comparison of clinical characteristics and environmental exposures between participants with and without paired serum for antibody testing for rickettsial diseases, Yangon General Hospital, Myanmar, 2015-2016**

| **Variable** | **Participants with paired serum N=367** | | **Participants without paired serum N=571** | |
| --- | --- | --- | --- | --- |
|  | **n** | **(%)** | **n** | **(%)** |
| **Demographics** |  |  |  |  |
| Age, median (range) years | 37 | (12-82) | 37 | (13-94) |
| Female sex | 172 | (46.9) | 267 | (46.8) |
| Rural | 267 | (72.8) | 433 | (75.8) |
| Presents during wet season^*^ | 208 | (56.7) | 313 | (54.8) |
| Income per month, Kyat^†^ |  |  |  |  |
| <100,000 | 148 | (40.3) | 211 | (37.0) |
| 100,000-300,000 | 172 | (46.9) | 272 | (47.6) |
| >300,000 | 47 | (12.8) | 88 | (15.4) |
| Agriculture worker | 24 | (6.5) | 50 | (8.8) |
| **Presenting symptoms** |  |  |  |  |
| Days unwell prior to presentation-median (range) | 7 | (1-365) | 7 | (1-180) |
| Days fever prior to presentation-median (range) | 5 | (1-30) | 5 | (1-30) |
| Chills and rigors | 174 | (47.4) | 249 | (43.6) |
| Cough | 103 | (28.1) | 176 | (30.8) |
| Headache | 168 | (45.8) | 232 | (40.6) |
| Fever ≥1 month | 73 | (29.9) | 117 | (20.5) |
| **Presenting signs** |  |  |  |  |
| Conjunctival suffusion | 9 | (2.5) | 13 | (2.3) |
| Eschar | 0 |  | 0 |  |
| Rash | 12 | (3.3) | 33 | (5.8) |
| Lymphadenopathy | 6 | (1.6) | 21 | (3.7) |
| Heart rate beats per minute, median (range) | 100 | (28-160) | 98 | (55-170) |
| Lung crepitations | 25 | (6.8) | 46 | (8.1) |
| Hepatomegaly | 15 | (4.1) | 36 | (6.3) |
| Splenomegaly | 11 | (3.0) | 13 | (2.3) |
| Meningism | 9 | (2.5) | 14 | (2.5) |
| qSOFA score ≥2^‡^ | 19 | (5.2) | 43 | (7.5) |
| **Environmental exposures** |  |  |  |  |
| Rodents around house | 70 | (19.1) | 164 | (28.7) |
| Rodent exposure at work | 11 | (3.0) | 29 | (5.1) |
| Rodent exposure last month | 15 | (4.1) | 29 | (5.1) |
| Livestock contact last month | 26 | (7.1) | 50 | (8.8) |
| Walking barefoot last month | 26 | (7.1) | 36 | (6.3) |
| Insect bite last month | 1 | (0.3) | 5 | (0.9) |
| Insect bite mark last month | 2 | (0.5) | 5 | (0.9) |

**NOTES:** *Wet season defined as May-October

†Equivalent in USD to <76.63, 73.63-220.89 and >220.89 in 2015[46]

‡qSOFA (quick SOFA) score: 1 point for GCS <15, Respiratory rate ≥22/minute, Systolic Blood Pressure ≤100

**Table S2. Comparison of clinical characteristics and environmental exposures between participants with and without PCR testing for rickettsial diseases, Yangon General Hospital, Myanmar, 2015-2016**

| **Variable** | **Participants PCR tested N=690** | | **Participants not PCR tested N=254** | |
| --- | --- | --- | --- | --- |
|  | **n** | **(%)** | **n** | **(%)** |
| **Demographics** |  |  |  |  |
| Age, median (range) years | 38.5 | (12-87) | 36 | (13-94) |
| Female sex | 353 | (51.2) | 147 | (57.9) |
| Rural | 411 | (59.6) | 112 | (44.1) |
| Presents during wet season^*^ | 532 | (77.1) | 172 | (67.7) |
| Income per month, Kyat^†^ |  |  |  |  |
| <100,000 | 266 | (38.6) | 96 | (37.8) |
| 100,000-300,000 | 325 | (47.1) | 122 | (48.0) |
| >300,000 | 99 | (14.4) | 36 | (14.2) |
| Agriculture worker | 46 | (6.7) | 28 | (11.0) |
| **Presenting symptoms** |  |  |  |  |
| Days unwell prior to presentation-median (range) | 7 | (1-365) | 7 | (1-365) |
| Days fever prior to presentation-median (range) | 5 | (1-30) | 5 | (1-30) |
| Chills and rigors | 298 | (43.2) | 127 | (50.0) |
| Cough | 201 | (29.1) | 79 | (31.1) |
| Headache | 292 | (42.3) | 110 | (43.3) |
| Fever ≥1 month | 127 | (18.4) | 64 | (25.2) |
| **Presenting signs** |  |  |  |  |
| Conjunctival suffusion | 16 | (2.3) | 6 | (2.4) |
| Eschar | 0 |  | 0 |  |
| Rash | 29 | (4.2) | 16 | (6.3) |
| Lymphadenopathy | 20 | (2.9) | 7 | (2.8) |
| Heart rate beats per minute, median (range) | 98 | (28-170) | 100 | (67-160) |
| Lung crepitations | 54 | (7.8) | 19 | (7.5) |
| Hepatomegaly | 30 | (4.4) | 22 | (8.7) |
| Splenomegaly | 18 | (2.6) | 6 | (2.4) |
| Meningism | 18 | (2.6) | 6 | (2.4) |
| qSOFA score ≥2^‡^ | 48 | (6.7) | 16 | (6.3) |
| **Environmental exposures** |  |  |  |  |
| Rodents around house | 148 | (21.5) | 87 | (34.3) |
| Rodent exposure at work | 22 | (3.2) | 18 | (7.1) |
| Rodent exposure last month | 24 | (3.5) | 21 | (8.3) |
| Livestock contact last month | 51 | (7.4) | 25 | (9.8) |
| Walking barefoot last month | 34 | (4.9) | 28 | (11.0) |
| Insect bite last month | 3 | (0.4) | 4 | (1.6) |
| Insect bite mark last month | 4 | (0.6) | 2 | (0.8) |

**NOTES:** Polymerase chain reaction: PCR

*Wet season defined as May-October

†Equivalent in USD to <76.63, 73.63-220.89 and >220.89 in 2015(64)

‡qSOFA (quick SOFA) score: 1 point for GCS <15, Respiratory rate ≥22/minute, Systolic Blood Pressure ≤100
